# Clinical effects of Ganglioside and fructose-1, 6-diphosphate on neonatal heart and brain injuries after Asphyxia

**DOI:** 10.12669/pjms.335.12830

**Published:** 2017

**Authors:** Xiaojing Zhu, Hongya Li, Congmin Zhang

**Affiliations:** 1 Xiaojing Zhu, Department of Pediatrics, First Center Hospital of Baoding, Baoding 071000, Hebei Province, P. R. China; 2 Hongya Li, Department of Pediatrics, First Center Hospital of Baoding, Baoding 071000, Hebei Province, P. R. China; 3 Congmin Zhang, Department of Pediatrics, First Center Hospital of Baoding, Baoding 071000, Hebei Province, P. R. China

**Keywords:** 6-Diphosphate, Asphyxia, Fructose-1, Ganglioside, Neonate

## Abstract

**Objective:**

To study the clinical effect of ganglioside (GM) and fructose-1, 6-diphosphate (FDP) on neonatal heart and brain injuries after asphyxia.

**Methods:**

Ninety-one neonates with asphyxia neonatal heart and brain injuries were randomly divided into an observation group and a control group. Both groups were given symptomatic treatment as soon as possible. On this basis, the observation group was given 200 mL of 5% glucose injection and 20 mg of GM and 250 mg/kg·d FDP by intravenous infusion. The above two drugs were given once a day for 14 days. The control group was given 20 mL of 5% glucose injection, 2 mL of cerebrolysin and 250 mg/kg·d FDP by intravenous infusion, once a day for 14 days. Both groups were administered on the first day after admission, and the course of treatment was 14 days. The treatment outcomes of the two groups were compared by detecting the levels of glycogen phosphorylase isoenzyme BB (GPBB), cTn-I and CK-MB, MRI results and Neonatal Behavioral Neurological Assessment (NBNA) scores before and after treatment.

**Results:**

The levels of GPBB, cTn-I and CK-MB in the observation group were significantly higher than those of normal neonates. After treatment, the levels of cTn-I and CK-MB in the observation group were closer to those of normal neonates compared with the control group, with significant differences (P<0.05). There was a significant difference in the brain MRI examination between the two groups (P<0.05). The NBNA scores of the two groups were significantly different before and after treatment (P<0.05). The total effective rate of the observation group was significantly higher than that of the control group (P<0.05).

**Conclusion:**

Neonatal heart and brain injuries after asphyxia can be well treated by combining GM with FDP.

## INTRODUCTION

Neonatal asphyxia, as one of the leading causes of neonatal death, is induced by hypoxemia, hypercapnia and metabolic acidosis for no spontaneous breathing or respiratory depression within one minute after delivery.^1^ Ischemia and hypoxia are the pathological basis and main manifestations after asphyxia, while the heart and brain are the important organs most vulnerable to damage.^2^ According to many years of experience in the treatment of neonatal asphyxia, we detected the levels of glycogen phosphorylase isoenzyme BB (GPBB), cTn-I and CK-MB that play important roles in heart and brain injuries. Meanwhile, MRI was also used to appropriately evaluate the degree of injury after asphyxia and to provide laboratory basis, so as to explore the clinical effect and feasibility of combining ganglioside (GM) with fructose 1, 6-diphosphate (FDP).

## METHODS

### Baseline clinical data

Ninety-one cases of neonatal heart and brain injury, which were admitted in our hospital from January 2014 to January 2015 and complied with diagnostic criteria for asphyxia, were selected. The neonates had the gestational ages of 34-42 weeks, with the birth weights of 1900-4100 g. There were 57 cases of normal delivery and 34 cases of cesarean section, including 53 boys and 38 girls. All neonates had no congenital malformation or acquired heart disease, and their mothers were all in good health. According to the Apgar score, there were 43 cases of mild asphyxia and 48 cases of severe asphyxia. The neonates were randomly divided into an observation group (n=52) (mild asphyxia (n=23), severe asphyxia (n=29)) and a control group (n=39) (mild asphyxia (n=19), severe asphyxia (n=20)). There were no significant differences in the gestational age, body weight, delivery mode, gender ratio or asphyxia degree between the two groups (P>0.05).

### Treatment methods

Both groups were given symptomatic treatment as soon as possible (including maintaining good ventilation, ventilating function, maintaining blood perfusion of all organs, maintaining blood glucose level normal, limiting fluid volume, controlling convulsions, reducing intracranial pressure, and maintaining water and electrolyte balances). On this basis, the observation group was given 200 mL of 5% glucose injection, 2 mL of cerebrolysin, 20 mg of GM and 250 mg/kg·d FDP by intravenous infusion. The above two drugs were given once a day for 14 days. The control group was given 20 mL of 5% glucose injection and 2 mL of cerebrolysin by intravenous infusion, once a day for 14 days. Both groups were administered on the first day after admission, and the course of treatment was 14 days.

### Observation indices and methods

The kits for detecting plasma GPBB, cTn-I and CK-MB were provided by ADL (USA). All selected neonates received chemiluminescence measurements. cTn-I was detected by Tosoh 360 chemiluminescence immunity analyzer (Japan) using a kit produced by Shanghai Ailex Technology Co., Ltd. (China) which was authorized by Tosoh, Japan) and its supporting reagents. GPBB was measured only once, but cTn-I was re-tested 2 weeks after treatment. CK-MB was detected by Hitachi 7180 biochemical analyzer (Japan) using a kit purchased from Shanghai Changzheng Co., Ltd. (China). After two weeks of treatment, the values of cTn-I and CK-MB in the neonates with asphyxia were examined again.

The blood samples of 54 healthy newborns delivered in our hospital, which were taken within 24 hour after birth, were selected to measure GPBB, cTn-I and CK-MB. The results were compared with those of neonates with asphyxia. The indices were detected again two weeks later. MRI was performed using Siemens 3T nuclear magnetic resonance equipment and re-tested two weeks after treatment.

### Evaluation of treatment outcomes

Excellent effects: breathing was stable within five days of treatment; clinical symptoms disappeared mostly or completely; convulsions were significantly alleviated or disappeared; consciousness turned clear; heart rate was greater than 110 beats/min, with strong heart sounds; the original reflex, limb muscle tension and ECG returned to normal; Neonatal Behavioral Neurological Assessment (NBNA) score >35. Good effects: breathing was stable within 10 days of treatment; clinical symptoms disappeared mostly or completely; consciousness turned clear; heart rate was relatively stable, greater than 100 beats/min, with strong heart sounds; original reflex could be partially elicited; limb muscle tension was improved; convulsions were alleviated significantly; ECG was significantly improved; NBNA score >35. No effect: consciousness was still not restored or the condition was exacerbated after over 10 days of treatment; all indicators were not improved; automatic discharge or death; NBNA <35.

### Statistical analysis

All data were analyzed by SPSS 12.0. The categorical data were expressed as mean ± standard deviation (x±s). The differences between groups and those before and after treatment were compared by the t test. The numerical data were subjected to the student’s t test.

## RESULTS

### Clinical therapeutic effects

After treatment, the observation group had 39 cases with excellent effects, 10 cases with good effects and three cases without any effects, and the control group and 13 cases with excellent effects, 17 cases with good effects and 9 cases without any effects. The total effective rate of the observation group was 94.2% (49/52) which was significantly higher than that of the control group (76.9%, 30/39) (P<0.05) (Table-I).

**Table-I:** Clinical therapeutic effects, case (%).

*Group*	*Excellent effect*	*Good effect*	*No effect*	*Total effective rate*
Observation (n=52)	39 (75.0)	10 (19.2)	3 (5.8)	49 (94.2)*
Control (n=39)	13 (33.3)	17 (43.6)	9 (23.1)	30 (76.9)

Compared with control group, *P<0.05.

### Levels of GPBB, cTn-I and CK-MB before and after treatment

The levels of GPBB, cTn-I and CK-MB in the observation group were significantly higher than those of normal neonates (P<0.01). After treatment, the levels of cTn-I and CK-MB in the observation group were closer to those of normal neonates compared with the control group, with significant differences (P<0.05) (Table-II and Table-III).

**Table-II:** Levels of GPBB, cTn-I and CK-MB before and after treatment in two groups (x±s).

*Group*	*Case No.*	*CGBB (ng/mL)*	*cTn-I (ng/mL)*		*CK-MB (U/L)*	

		*Before Treatment*	*Before Treatment*	*After Treatment*	*Before Treatment*	*After Treatment*
Observation	52	16.81±4.22**	1.40±0.18**	0.36±0.11^Δ^	48.12±13.48**	22.25±4.53^Δ^
Control	39	15.39±4.27**	1.49±0.21**	0.56±0.14	44.31±9.78**	28.36±5.42
Normal neonates	54	4.28±2.78	0.24±0.05		17.32±6.41	
t		9.31;8.08	11.6;7.98	7.76	4.33;4.92	7.12

** P<0.01, compared with normal neonates

Δ P<0.05, compared with control group.

**Table-III:** Levels of GPBB, cTn-I and CK-MB before and after treatment according to degree of asphyxia (x±s).

*Group*	*Case No.*	*CGBB (ng/mL)*	*cTn-I (ng/mL)*		*CK-MB (U/L)*	

		*Before Treatment*	*Before Treatment*	*After Treatment*	*Before Treatment*	*After Treatment*
Observation	48	29.62±5.31**	2.24±0.25**	0.71±0.21^Δ^	68.08±12.58**	29.25±7.35^Δ^
Control	43	12.38±4.58**	0.91±0.18**	0.35±0.15	39.32±10.32**	21.65±5.89
Normal neonates	54	4.29±2.87	0.21±0.07	17.35±6.54		
t		10.45;4.56	12.15;5.11	5.36	7.05;5.61	4.96

** P<0.01, compared with normal neonates

Δ P<0.05, compared with control group.

### MRI results before and after treatment

The observation group had 14 cases of subarachnoid hemorrhage, four cases of subdural hemorrhage, 2 cases of intracerebral hemorrhage and one case of intraventricular hemorrhage. The control group had 12 cases of subarachnoid hemorrhage, three cases of subdural hemorrhage, one case of intracerebral hemorrhage and one case of intraventricular hemorrhage. MRI discloses that 50 of the 52 cases in the observation group recovered to normal, and 34 cases in the control group basically recovered. There was a significant difference in the brain MRI examination between the two groups (P<0.05).

### NBNA scores before and after treatment

The NBNA scores of the two groups two and four weeks after treatment were significantly higher than those before treatment (P<0.05) (Table-IV).

**Table-IV:** NBNA scores before and after treatment (x±s).

*Group*	*Case No.*	*Before Treatment*	*2 Weeks After Treatment*	*4 Weeks After Treatment*
Observation	52	25.43±4.52	37.98±4.09**^Δ^	40.78±4.89**^Δ^
Control	39	27.32±5.58	33.08±3.23**	35.52±3.82**

** P<0.01, compared with normal neonates

Δ P<0.05, compared with control group.

## DISCUSSION

Asphyxia is the most common neonatal disease and also one of the main reasons for neonatal disability and death. It is reported in the literature that the incidence of the disease is 3%-12% in developing countries, which is 4.7%-8.9% according to domestic reports.^3^ Neonatal hypoxia can cause systemic multiple organ damage, of which the incidence of organ damage is 74.8%, 65.3% for brain damage and 20%-37.8% for myocardial injury.^4^ The disability and mortality caused by multiple organ damage for asphyxia may be up to 30%. Ischemia and hypoxia is the main manifestation and pathological basis of asphyxia, and the heart and brain are the important organs the most vulnerable to damage, therefore, it is of great significance to make early diagnosis and timely treatment on neonatal asphyxia. Hypoxic ischemic brain damage is a neonatal brain injury caused by fetal distress and neonatal asphyxia, with a high disability and mortality. It is also a major cause of later mental retardation and cerebral palsy. Therefore, great attention has been paid in clinical work to the harm, treatment and prognosis of brain injury, which has also achieved a certain efficacy. After treatment, the therapeutic effect of brain injury after asphyxia can be improved. Cardiac dysfunction is also an important part of multiple organ damage after asphyxia, which is easily overlooked due to no obvious specific clinical manifestations. The damage of neonatal heart function caused by asphyxia is often a key factor in determining the changes in infants’ conditions.^5^ If the damage is not detected early and given effective treatment timely, the mortality of the infants will be significantly increased. Therefore, how to take effective prevention and early treatment, shorten the time of ischemia and hypoxia, improve the prognosis and the quality of life of infants has become a crucial problem to be resolved by clinical pediatricians.

The results showed that the levels of GPBB, cTn-I and CK-MB were the highest in the severe asphyxia group, and the values of the mild asphyxia group were lower compared with the former group, but the values of both groups were higher than those of normal neonates. It suggested that the above indicators could be used as a basis for early diagnosis of heart and brain damage and prognosis. After treatment, the values of cTn-I and CK-MB were decreased significantly in both the groups (P<0.01). And compared with the control group, the indicators of the observation group after treatment were closer to those of the normal neonates, with the differences statistically significant (P<0.05). As GPBB is highly specific and sensitive in the early stage of heart and brain injury, it is close to the normal range after 24h-36h, so the value of GPBB was only observed before treatment. cTn-I, as a cardiac-specific antigen, is a marker of sensitivity and high specificity of myocardial injury.^6^ CTn-I do not exist in skeletal muscle, but only exists in the myocardial cells, so it is an ideal cardiomyocyte-specific marker. Due to hypoxia, the biofilm of myocardial cells is damaged, and cTn-I free in the cytoplasm can be quickly released into the blood circulation in the early stage of myocardial cell injury. The level of serum cTn-I was increased in three to five hours, followed by continuous disruption and destruction of myofibril, and cTn-I was constantly released, therefore, cTn-I is a serum marker of high sensitivity and specificity for reflection of myocardial cell damage.^7^ A previous study showed both serum cTn-I and CK-MB were significantly increased on the first day of neonatal asphyxia, showing a positive correlation.^8^ Therefore, the indicators are of great significance to the diagnosis of myocardial damage and judgment of efficacy and prognosis. CK-MB mainly exists in the cytoplasm of myocardial cells, with a relatively good specificity. The pathological changes of myocardial damage caused by neonatal asphyxia are mainly that hypoxia, ischemia, acidosis and hypercapnia can directly damage myocardial cells, leading to myocardial cell hypoxia and ischemia, cell membrane damage, enzyme spillover from myocardial cells and elevated serum levels of myocardium enzymes. GPBB is an early biochemical marker found in recent years that can reflect the hypoxia and ischemia of brain cells and myocardial cells. Existing in human brain and myocardial cells in a large amount, it is a key enzyme of glycogen breakdown during oxidative phosphorylation of myocardial cells and also the most sensitive and specific component of myocardial and brain cells on hypoxia and ischemia.^9^ Under normal conditions, GPBB can tightly integrate with the sarcoplasmic reticulum in myocardial cells in the form of GPBB glycogen complex, which cannot be broken down easily. During hypoxia and ischemia of myocardial cells, mitochondrial oxidative phosphorylation is blocked, to mobilize glycogen breakdown for energy supply, so that the combined GPBB transforms from a binding type to a free one, and then enters the peripheral blood with the increase of cell membrane permeability. With the damage of myocardium, accompanied by exacerbation of the disease, nerve cells are damaged and blood-brain barrier is broken after brain damage, and GPBB in the brain tissue is also released into blood, becoming a basis for brain injury and treatment. This study showed that the increased degree of GPBB was positively correlated to the degree of asphyxia.

### The value of diagnosis and treatment of asphyxia brain injury

Brain injury after asphyxia is one of the important causes of high disability of newborns. Given its short window phase of treatment, early diagnosis is the key to treatment. Brain injury in the nervous system often appears 12-36 h after birth, which is difficult to be diagnosed just relying on clinical manifestations. MRI is superior to CT in assessing the nature and extent of brain damage. It has the advantages of multi-axial imaging, high resolution and no radioactive damage. It is particularly sensitive to the diagnosis of sagittal parabrachial and basal nucleus lesions, and the lesions can be displayed as a high signal on the first day after delivery, therefore, MRI is more convincing to be taken as a basis for the diagnosis of brain injury and observation of efficacy.

In this study, the brain MRI results showed that 50 cases returned to normal in the observation group and 34 cases in the control group, with a significant difference (P<0.05), indicating that the efficacy of the observation group on neonatal asphyxia is superior to that of the control group.

The results showed that all the performance indicators of the observation group were better than those of the control group, between which the difference was statistically significant, suggesting that the combined application of GM and FDP has a clinical value on the treatment of heart and brain injury after asphyxia. The synergistic use of the two drugs can take remarkable curative effects on the protection of ganglion cells and myocardial cells, inhibition of apoptosis and improvement of heart and brain damage after hypoxia and ischemia.

As apoptosis is involved in the process of heart and brain injury after asphyxia, so how to inhibit and reduce apoptosis is of vital importance to improve the quality of life after asphyxia rescue and reduce mortality and morbidity. It has been shown that GM has a direct inhibitory effect on apoptosis. GM, as a component of most mammalian cell membranes, is abundant in the central nervous system, especially in the gray matter of the brain, which, therefore, gets high attention to. GM plays an important role in the differentiation and development of neurons and axons, the repair of nerve tissue and the plasticity of neurons. Exogenous GM can easily pass through the blood-brain barrier, embed in the nervous cell membrane structure, stimulate the potential compensatory mechanism after the central nervous system injury, maintain the balance of ions inside and outside the membrane, reduce K^+^ influx and Ca^2+^ overload, and alleviate cerebral edema and other pathological damages. It takes effects by controlling the expression of apoptotic genes, thereby preventing the damage from deepening, protecting unimpaired nerve cells, and helping the long-term learning and memory ability after injury, so as to achieve an effective therapeutic effect.

FDP is a kind of substance with efficient energy supply. As a buffer of energy and an energy carrier in tissues, it has significant protective effects on ischemic myocardial contractile function. In case of myocardial ischemia, it can convert ADP to ATP to provide the reserve for maintaining myocardial energy, reduce the formation of oxygen free radicals, increase membrane stability, and protect the cell membrane from the invasion of radicals and other harmful substances. In addition, it can also inhibit membrane permeability through the cell membrane, change the opening of the channel, protect mitochondria, reduce damage to the cell membrane, and reduce the probability of apoptosis caused by myocardial ischemia and hypoxia.

### Conclusion

In conclusion, this study showed that the combined therapy of GM and FDP in the treatment of heart and brain injury after asphyxia has significant protective effects on ganglion cells and myocardial cells. The detection on the indicators such as MRI, cTn-I and CK-MB before and after asphyxia respectively found that the therapy could significantly improve the success rate of rescue and reduce the disability, and the observed objects of the two groups had no significant adverse reactions during treatment, and all could adhere to clinical treatment and observation. It indicates that the drug treatment of neonatal diseases has a safe and significant effect, with extensive prospects of application and popularization. It can significantly improve the level of diagnosis and treatment of such kind of disease, which is worth promoting in primary hospitals.

### Authors’ Contributions

**XZ** designed this study and prepared this manuscript. **XZ, HL & CZ** performed this study. **HL & CZ** collected and analyzed clinical data.
